# Competency in Navigating Arbitrary Spaces as an Invariant for Analyzing Cognition in Diverse Embodiments

**DOI:** 10.3390/e24060819

**Published:** 2022-06-12

**Authors:** Chris Fields, Michael Levin

**Affiliations:** 1 Allen Discovery Center at Tufts University, Science and Engineering Complex, 200 College Ave., Medford, MA 02155, USA; fieldsres@gmail.com; 2 Wyss Institute for Biologically Inspired Engineering at Harvard University, Boston, MA 02115, USA

**Keywords:** physiology, anatomical morphospace, basal cognition

## Abstract

One of the most salient features of life is its capacity to handle novelty and namely to thrive and adapt to new circumstances and changes in both the environment and internal components. An understanding of this capacity is central to several fields: the evolution of form and function, the design of effective strategies for biomedicine, and the creation of novel life forms via chimeric and bioengineering technologies. Here, we review instructive examples of living organisms solving diverse problems and propose competent navigation in arbitrary spaces as an invariant for thinking about the scaling of cognition during evolution. We argue that our innate capacity to recognize agency and intelligence in unfamiliar guises lags far behind our ability to detect it in familiar behavioral contexts. The multi-scale competency of life is essential to adaptive function, potentiating evolution and providing strategies for top-down control (not micromanagement) to address complex disease and injury. We propose an observer-focused viewpoint that is agnostic about scale and implementation, illustrating how evolution pivoted similar strategies to explore and exploit metabolic, transcriptional, morphological, and finally 3D motion spaces. By generalizing the concept of behavior, we gain novel perspectives on evolution, strategies for system-level biomedical interventions, and the construction of bioengineered intelligences. This framework is a first step toward relating to intelligence in highly unfamiliar embodiments, which will be essential for progress in artificial intelligence and regenerative medicine and for thriving in a world increasingly populated by synthetic, bio-robotic, and hybrid beings.

*“Intelligence is a fixed goal with variable means of achieving it.”*

—William James

## 1. Introduction

Perhaps the most striking property of life, when contrasted with inanimate objects and the artifacts of human engineering to date, is its ability to operate adaptively in a range of problem domains. This adaptability persists, as noted by James in the quotation above [1], even when circumstances require qualitatively different adaptive responses. Living systems at all scales—from cells to swarms of organisms—exhibit preferences about specific states and exert energy to achieve those states by any means available. There is great variety in the degree of adaptive competency seen across the biosphere, ranging from simple homeostatic processes to complex minds with meta-cognition able to not only pursue complex goals but to set and reset those goals [2]. The capacity to navigate and behave in three-dimensional space via degrees of memory, foresight, creativity, etc. has been long studied by behavioral and cognitive science. More recently, the capacities of humans and other animals to navigate and behave in complex social environments has also been intensively investigated, as has the ability of humans (infants and adults) and other animals to detect intelligent agents in their environments and form a theory of mind [3]. However, the omics revolution and the availability of big data in multiple domains, as well as the emerging fields of basal cognition, synthetic bioengineering, and artificial intelligence, require the development of novel frameworks for modeling “navigation” and “behavior” in abstract “spaces” at multiple scales and for understanding the relationships between them.

It is now clear, for example, that molecular networks, single-celled organisms, tissues, and organs exhibit behaviors that, when viewed at a suitable level of abstraction, can be placed on the same continuum as familiar model systems studied in neuroscience [4,5,6,7,8,9,10]. The conservation of molecular mechanisms supporting behavioral functionality is now being characterized in the field of basal cognition, making it clear that flexible, adaptive behavioral competencies long predate the appearance of complex brains. Moreover, a wide range of novel organisms including cyborgs, hybrots, biobots, and others are being created by chimeric approaches that combine evolved and designed material. These efforts give rise to beings that cannot be placed within the natural phylogenetic tree of Earth, with behavioral competencies that cannot be readily guessed by analogy to familiar forms selected within specific environments [11,12,13,14,15,16,17,18,19,20,21,22].

**Figure 1:**
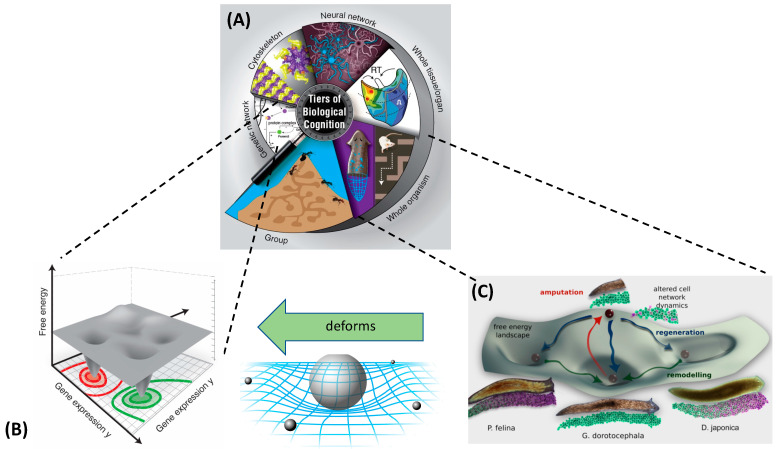
Multi-scale competency architecture (MCA). (**A**) The MCA is implemented by biological systems in which every level of organization traverses various spaces toward preferred regions. Subcellular systems (molecular networks) navigate the transcriptional space (**B**), while collections of cells navigate the anatomical morphospace, such as planarian tissues that can be pushed into regions of the space corresponding to diverse species’ head shapes without genomic editing (**C**) (image by Alexis Pietak [31]). Higher-order systems distort the energy landscapes for their subsystems (via virtual “objects” in that space) to enable their components’ local homeostatic mechanisms to achieve goals that are adaptive at the higher level systems’ space. This links the intelligence (or competent navigation) of spaces to simple energy minimization dynamics. Panels (**A**,**B**) are courtesy of Jeremy Guay of Peregrine Creative.

The boundaries between “organisms” and “machines” are, moreover, rapidly disappearing [23] as evolutionary techniques are used by machines to create other machines, and biological control systems become increasingly tractable to reprogramming [24,25]. These advances suggest that the classical definitions of intelligence, agency, cognition, and similar terms, based on the limitations of technology and imagination, are unlikely to survive the next few decades. It is essential now to develop frameworks that generalize across the space of possible beings and focus not on the contingent facts of a creature’s composition or provenance (e.g., evolved vs. designed) but rather on deep functional aspects. We must learn to recognize, repair, create, and relate to novel beings, with minds of diverse cognitive capacity in new and unfamiliar forms. While we (and many other animals) are very good at recognizing agency in both the three-dimensional world of conventional behavior and the much higher dimensional space of social interactions, we are poor at recognizing intelligence in novel guises. Hence, we often neglect the intelligence underlying competencies at the sub-organismal scales (Figure 1A). This acts as a brake on technological progress (in robotics and in biomedical science) and holds back the development of new systems of ethics that are required for a world outside of a Garden of Eden in which we would be confronted only by a finite, unchanging set of standard animals. Toward the development of mature theories of intelligence based on cybernetic principles and not frozen accidents of the evolutionary stream on Earth, we propose a framework—one based on the well-established ideas of hierarchical Bayesian active inference [26,27,28,29,30]—that generalizes the notion of the ”space” within which an agent can operate and defines intelligence as the competency of navigating that space. Our goal is to identify a deep invariant that would be useful across truly diverse intelligences and that would establish a rigorous conceptual basis to advance empirical studies of agency across embodiments. As we show in what follows, minimization of the Bayesian prediction error—cast formally as minimization of a variational free energy (VFE) [26,27,28,29,30]—meets these requirements.

Understanding how agents of all kinds—from evolved natural forms to bioengineered creations—solve problems is of high importance on several fronts. From the basic science perspective, we stand to gain a more profound understanding of the evolutionary process and how it innovates [32,33]. Fascinating questions surround the relationship of biological hardware determined by DNA and the dynamic functionality upon which selection operates. There is also a set of practical impacts. Biomedicine risks stagnating in the low-hanging fruit of single-gene and single-cell diseases that are reachable by genomic editing and stem cell biology without exploiting the “software” of life [34]. Understanding the algorithms that enable life to thrive despite a wide range of perturbations (described below) offers a roadmap for regenerative medicine in which we exploit the competencies of cellular collectives to achieve system-level outcomes that are simply too complex to micromanage [35,36]. Micromanaging complex systems is very difficult and faces intractable inverse problems [37] with respect to knowing what pathway or gene to edit to achieve, for example, organ regeneration. A mature understanding of how biology at all scales solves problems could enable bioengineers to work in much simpler, lower-dimensional spaces in which we identify triggers or stimuli for top-down control of form and function. Top-down control of decision making at the organ level avails regenerative medicine of the master triggers that affect a kind of behavior shaping of body cells and tissues to induce predictable changes in growth and form that are too hard to force from the bottom up [35,36,38].

The philosophical basis of our perspective has been described previously; it dates back at least to Ashby [39] and was featured prominently in the work of Maturana and Varela [40], Pattee [41], and Rosen [42,43], among many others. It is fundamentally an observer-focused, gradualist, substrate-independent view of agency that takes evolution and developmental biology seriously. We focus on embodied, enactive cognition, on “life as it can be”, on the processing of information by agents that exist at multiple scales in living organisms, and on goal-directed activity within an active-inference framework. We generalize “behavior” to include actions in diverse problem spaces and focus on the role of the observer in defining “problems” and “strategies (of various levels of sophistication)” as hypotheses to be evaluated based on the degree of prediction and control they provide. We emphasize that estimates of the intelligence of any system, natural or not, are fundamentally an IQ test for the observer, requiring us to acknowledge our own limitations in being able to detect intelligent functionality that differs from our own in embodiment or in its goals. This viewpoint was for example the basis for the well-known Turing test. Thus, we seek a framework that is general and constrained as little as possible by parochial assumptions of the standard human experience of intelligent behavior limited to medium-sized, medium-speed objects operating in the 3D world accessible to direct visual perception.

In what follows, we first review the ubiquitous use of abstract spaces to organize observed biological behavior. We show how, in every case, the biological systems in question can be seen as actively behaving in the relevant space. We discuss, in particular, examples of living systems solving problems in transcriptional, physiological, and anatomical spaces. From this perspective, development and regeneration are the result of a collective intelligence of cells navigating the anatomical morphospace, and devices such as pacemakers and insulin pumps, as well as our standard organs, become more complex versions of Braitenberg-like “vehicles” [44] navigating in physiological space. A key communication mechanism enabling this collective intelligence is resource exchange (e.g., a cell or tissue serving as memory in exchange for food). Hence, it is possible to use “economic” thinking to understand homeo- or allostasis. We show how these ideas generalize across multiple spaces of interest and suggest that a relatively small number of mechanisms organize behavior in any “space” of biological significance.

We generalize from such examples to establish the notion of an arbitrary space in which agents operate and in which components distort the spaces for their subcomponents, causing them to traverse geodesics with adaptive consequences for the higher levels (Figure 1B). This facilitates rigorous recognition, comparison, and manipulation of agents’ behaviors. We redefine “environment” to mean not just external objects but the internal components that serve as an environment to the inner modules that cooperate and compete within and across the levels of organization. We propose an account of what spaces are, how they come to be, and how observers, selves, and agents operate in those spaces, complementing the notion of empowerment from robotics [45,46,47,48,49,50,51,52,53,54]. We formalize the notion of an observer’s reference frame and show how fundamental active inference processes give rise to an abstract idea of an agent’s “action space” that abstracts away from any particular set of degrees of freedom and hence allows “action” in spaces characterized by, for example, transcriptional or morphological degrees of freedom, as well as in ordinary 3D space with its position and momentum degrees of freedom. Our highly integrative framework shows how information exchange (mutual mechanistic constraints) between spaces and scales enables sophisticated, adaptive system-level computation and efficient representation. We end with a discussion of the implications of, as well as the new research directions made possible by, this new lens with which to see how agents represent their worlds.

## 2. Abstract Spaces Reveal Behavior across Biology

A ‘space” is just a collection of states, together with some notion of similarity or “distance” between states. The use of omics technologies has made commonplace the ideas of the “states” of the genome, transcriptome, proteome, and metabolome. Statistical measures of the similarity of such states are often based on the assumption that such states form a space. Counting the number of base differences between two DNA or RNA sequences or of amino acid or amino acid family differences in polypeptide sequences provides a well-known example (e.g., see [55] for an early investigation of metrics of this kind). Adopting the formalism of a space allows the use of dynamical concepts, such as attractors and flows, as well as the concepts of similarity and distance [56].

While the spaces defined by omics technologies are often regarded as theoretical constructs that we, as external observers, employ to organize and analyze data, it is equally natural to regard these as spaces that the system of interest traverses. We suggest abandoning the concept of a single, objective “behavior” that a system is “really” performing in favor of an abstract “action space” that incorporates all of the ways in which an organism can manipulate its own or its environment’s degrees of freedom. We propose an observer-centered view in which statements about goals and cognitive properties are, in effect, engineering claims to be empirically evaluated based on how much progress and control they drive. Thus, in place of Morgan’s Canon [57], which urges erring on the side of underestimating the level of agency in systems (a kind of scientific mind blindness as an a priori preference), we propose an unbiased view in which multiple observers’ proposed problem spaces and levels of competency within those problem spaces could be equally useful.

In this framework, “preferred” homeostatic or allostatic states become attractors for the dynamics executed by the system [58,59,60,61]. Within the hierarchical Bayesian active inference formalism, they are states that minimize variational free energy (VFE) and hence maximize the probability of correct predictions as discussed below. An early insight of this approach [27] is that minimizing VFE requires probing the environment to determine how it behaves when actively perturbed. While this notion of *active* inference, with its connotation of agency, seems obvious for complex organisms feeding, fighting, fleeing, or engaging in social behaviors, we will see below that it is theoretically productive from the scales of cells and tissues to those of communities and ecosystems [62,63].

## 3. Transcriptional, Metabolic, and Physiological Spaces

**Figure 2:**
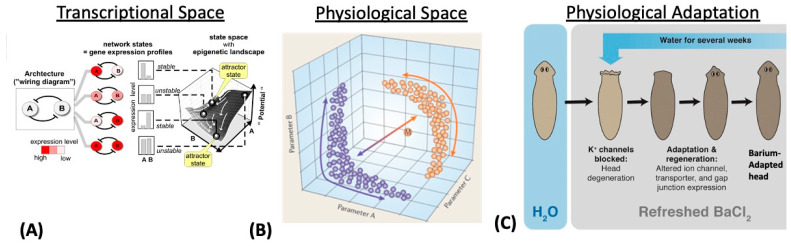
Diverse spaces within which living systems navigate. Transcriptional space is the space of possible gene expression patterns, taken with permission from [59]. Examples of a transcriptional state space of a two-gene network (mutual inhibition of genes (**A**,**B**)) and the associated epigenetic landscape in the two-dimensional state space are shown. The dynamical state of the network maps to a point in the space; changes in gene activity represent walks in the space. (**B**) Physiological space is the space of possible physiological states, simplified to just three parameters such as intracellular ion concentrations, taken with permission from [70]. Individual cells occupy regions of the space and can move between states by opening and closing specific ion channels. The functional state (region of the space) is a function of all of the parameters and large-scale variables, such as V_mem_ (resting potential), which refer to numerous microstates composed of individual ion levels. (**C**) Navigating spaces to thrive despite novel stressors, taken with permission from [71]. Planaria exposed to barium chloride experience head deprogression because barium is a blocker of potassium channels, making it impossible for the neural tissues in the head to maintain a normal physiology. The flatworms soon regenerate a new head which is barium-insensitive. Transcriptomic analysis showed only a handful of genes whose expression was altered in the barium-adapted heads. Because barium is not something planaria encounter in the wild, this example shows the ability of the cells to navigate transcriptional space to identify a set of genes that enable them to resolve a novel physiological stressor. The mechanism by which they rapidly determine which of many thousands of genes should be up- or downregulated in this scenario is not understood. (The cells do not turn over fast enough to allow a hill-climbing search, for example.)

Some cell-level capabilities have only been considered traversals of “spaces” since the development of omics technology and big databases. Being able to look at a transcriptome, for example, over time made the idea of a transcriptional space—a space of all possible gene expression patterns—obvious. However, we still have not, as a research community, begun to view the transition of the system through the space as active navigation with the cell as an agent. It is usually thought to be a descriptive view of a physical process (although, see the work on dynamic adaptation in physiological space [64,65,66] and on navigation of biochemical networks space [67]). Control systems acting on the transcriptome, proteome, and metabolome or on the “interactome” that spans all three are typically thought of as mechanisms and not as information-processing systems that display active intelligence. (However, see [68], in which metabolite regulation of metabolic pathways is characterized as “heuristic”, and [69], in which the consequences of metabolic decisions are considered in development and disease.) These and other biological phenomena can, however, be cast as behavior and problem solving in appropriate spaces, such as the transcriptional and physiological spaces depicted in Figure 2A,B, respectively. Doing so allows all of the tools of cybernetics, control theory, and the cognitive and behavioral sciences to be brought to bear. From this perspective, physiological prosthetics (such as implanted smart insulin or neurotransmitter pumps) are simple robots navigating a problem space.

As an example of problem solving in unconventional spaces, consider the following. When planarian flatworms are exposed to barium, a non-specific blocker of potassium channels, their heads rapidly degenerate due to the stress of the neural tissues’ inability to regulate the ionic balance (Figure 2C). Remarkably, when kept in a barium solution, the remaining tails regenerate new heads which are barium-insensitive [71]. Transcriptomic analysis reveals that the difference between the wild-type and barium-adapted heads occurs in only a handful of transcripts. The key facts are that barium is not something that planaria encounter in the wild (thus, there is not a selective pressure to specifically evolve responses to this toxin) and that planarian cells do not turn over fast enough to employ a bacteria-like selection mechanism (random change to test all possible transcriptional responses, with a rare survivor clone repopulating the head). How, among the very high-dimensional space of all possible gene expression levels, do the cells know exactly which small complement of transcriptional responses is needed to solve this physiological stressor? There is no time to try every possible combination (which would be astronomical, and many of which would kill the cell anyway).

This problem can be formulated as a search policy for navigating transcriptional space (i.e., the space for some specific organism of all possible gene expression patterns). Similar problems have been shown in other model systems of developmental robustness, suggesting exploration strategies that avail organisms of rapid, Lamarckian-like adaptation to stress and changing environmental conditions [72,73,74,75]. It is not known yet how the planaria do this, but one possibility involves generalization (one dimension of intelligence) to recognize barium-induced physiological states as belonging to a class of other problems (like excitotoxicity) for which planarian cells may have evolved solutions. Perhaps, like bacterial metabolism sensing systems [68,76,77], the cells detect (and act on) highly processed state information several steps removed from the proximal events at the membrane. In this case, the many ways to depolarize tissue could be naturally coarse-grained to represent a single problem: a change in membrane voltage addressable by a single set of transcriptional actions. As shown in [78], acting on such coarse-grained information is a simple form of meta-processing (i.e., “higher-level” information processing that controls some lower-level process). The planarian’s response to barium can, from this perspective, be seen as a very primitive form of meta-cognition.

## 4. Morphospace: Control of Growth and Form as a Collective Intelligence

**Figure 3:**
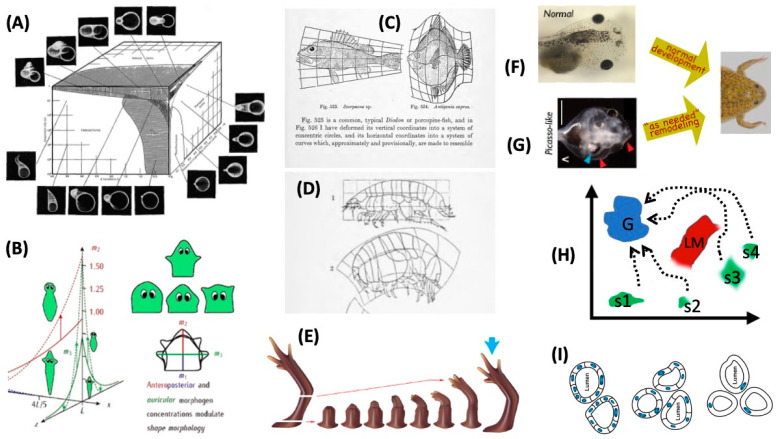
Anatomical morphospace and its navigation by cellular collective intelligence. (**A**) Example of morphospace—the space of possible shapes—for coiled shells (taken with permission from [87]). Three parameters (rate of increase in the size of the generated shell cross section per revolution, the distance between the cross section and the coiling axis, and the rate of translation of the cross section along the axis per revolution) define a space within which many taxa can be found. (**B**) Space of possible planarian heads defined by possible values of three morphogen values in a computational model (taken with permission from [88]). (**C**,**D**) The idea of morphospace and different species of animals as mathematical transformations of coordinates in those spaces was originally proposed by D’Arcy Thompson (panels taken with permission from [89]). Traversals of morphospace can be seen in regeneration, such as for the salamander limb, which will continue to grow when amputated at any position (brought to a new region of morphospace for the limb) until the system reaches the correct state (the shape of a normal limb), at which point it stops ((**E**) panel by Jeremy Guay of Peregrine Creative), or in the ability of both normal and scrambled tadpole faces to rearrange until a correct frog craniofacial morphology is reached ((**F**,**G**) taken from [90]). (**H**) Remodeling, de novo embryogenesis, and regeneration are all examples of biological systems’ abilities to navigate from starting positions in morphospace “s1”–“s4” and reach the target morphology goal state “G” while avoiding the local maxima “LM”. Morphospace plasticity (**I**) includes the ability of higher-level constraints to activate diverse underlying molecular mechanisms as needed. For example, (**I**) tubulogenesis in the amphibian kidney normally works via cell–cell communication, but when the cells are forced to be very large (by induced polyploidy), this reduces the number of cells and eventually leads to switching to using cytoskeletal bending to form the same diameter of tube from just one cell bending around itself (panel by Jeremy Guay from [91,92]).

A key aspect of formulating intelligence-based models is the recognition that all intelligent agents are *collective* intelligences; their problem-solving capacities rely on the competencies of their parts and the architecture of their relations. Much like how individual cells’ capabilities rely in part on gene-regulatory networks, which also have the capacity for learning [79,80,81,82,83], multicellular creatures have to rely on cellular behaviors in order to achieve goals in the anatomical morphospace, or the space of possible shape configurations [84,85,86]. One way to begin to understand the scaling of intelligence toward higher-level goals is to consider how the collective intelligence of cellular swarms implements large-scale anatomical homeostasis. Panels A–I in Figure 3 illustrate a number of examples of swarm intelligence as the ability to reliably navigate to a correct target morphology (region of the morphospace) despite perturbations or changing starting positions.

During embryonic development, each of us recapitulates evolution’s journey across the Cartesian cut: we begin life as a single cell (the fertilized egg), which replicates and eventually self-assembles a complex and sometimes highly cognitive being. This process is often presented as a feedforward emergence of complexity via massively parallel local rules, and much progress in molecular genetics has shed light on the subcellular hardware necessary for it to occur. The developmental morphogenetic field concept anticipated some aspects of this framework, although the mechanistic information linking the parameters defining movements in the space and the mediator of the information field is only becoming apparent now [86,88,93,94,95,96,97,98,99]. What is only now beginning to be rigorously understood is the degree of intelligence, in William James’s sense, of this process. It is reliable but not hardwired. It exhibits remarkable stability and robustness, capable of reaching the same target morphologies despite significant departures from evolutionarily expected (default, wild-type) components and environmental conditions.

One potential source of the targeted plasticity of embryonic development is that development is, from the very first zygotic division, a process of communication and negotiation. In some organisms (e.g., *Caenorhabditis elegans*), the first division establishes the anterior-posterior axis, an asymmetry encoded by differences in the protein and RNA content of the first-division daughter cells [100]. In others (e.g., *Xenopus laevis*), bioelectric asymmetry at the first division (driven by cytoskeletal symmetry breaking) establishes the left-right axis [101,102]. As soon as cells have some distinction, they have something to communicate about. This can be understood in terms of VFE minimization, as described further below. The behavior of a cellular neighbor with distinct properties is not as easy to predict compared with that of an identical neighbor [103]. The cells on the exterior of an embryo, for example, are more exposed to the environment than the interior cells; hence, they are responsible for both protecting and, in the absence of a yolk sac, feeding the interior cells. What interior cells provide in return is, in many cases, information [104], with neurons as the evolutionary specialists for this task. This kind of communication-dependent specialization, and hence the division of labor in building the embryo and then the adult organism, obviously suggests economic exchange, as well as deception, coercion, and other strategies employed in organism-scale social relations [105], with phenomena such as cellular cytotoxicity [106] and the cells’ ability to gauge the fitness of their neighbors [107,108] as the ultimate “policing” actions.

It is clear that cooperation and competition [109] among cells reliably results in complex target morphologies during embryogenesis. Indeed, this capability is a sought-after capacity in swarm robotics [110]. Importantly, robotics, neuroscience, and the field of collective intelligence [111,112] all focus on the ability of swarm dynamics to give rise to emergent minds. How much and what kind of intelligence could a society of cells exhibit? Evidence for goal-directed behavior (a hallmark of a coherent intelligence in any embodiment), including the sophisticated ability to achieve those goals despite unexpected circumstances (activity beyond fixed responses), abound in developmental and regenerative biology. Morphogenesis is extremely tolerant to novelty not just in changes to the external environment but changes in its own components via natural mutation or engineering.

One example is the regenerative properties of animals like planaria and axolotls [113]. When a salamander limb is amputated at any level, the cells will rapidly grow and remake a limb, stopping *when the correct limb is completed*. The collective pursues this goal from diverse starting positions and executes a test-operate-exit loop [36] to deal with unpredictable types of damage. Some progress has been made on the mechanisms that serve as the cognitive glue binding individual competent cells together into an emergent collective intelligence that can operate toward an outcome far larger than any individual cell (i.e., only defined at the system scale). Perhaps unsurprisingly (but only in retrospect), this involves preneural bioelectric signaling, where cells form bioelectric networks that scale [9,114,115] individual cell capacities toward larger (anatomical) goals. By perturbing this system, not only can the pattern memories of the collective intelligence be altered (for example, permanently changing the number of heads that genetically wild-type planarian tissues consider to be their correct target morphology) [116,117], but they can be pushed into the regions of an anatomical state space belonging to *other species*. In planaria, temporary disruption of bioelectrical connectivity among cells (with no genomic editing) leads to the regeneration of heads (including brain shape and stem cell distribution) appropriate to other extant species of planaria [31,118], which are 100–150 million years apart phylogenetically.

The ability to repair damage toward specific configurations in the anatomical morphospace is not only seen in adult regeneration, since regulative development allows bisected embryos to make normal monozygotic twins, and it compensates for huge changes in the number of cells during development [119,120]. Another example concerns the conversion of tadpoles into frogs by the movement of craniofacial organs. It was found that this is not a hardwired process where each organ simply moves a predetermined amount in the right direction. When tadpoles with scrambled faces are created, they change into largely normal frogs [90,121,122], because the eyes, jaws, etc. move in novel directions and across new paths (in fact sometimes overshooting and coming back to the correct positions) to form a correct frog face. These capacities in development, regeneration, and metamorphosis can all be seen as diverse examples of one basic underlying capacity: anatomical homeostasis (error reduction loop with respect to the metrics in the anatomical space), which requires policies for actions which reduce the delta from the current state and target state. In most cases (including the frog face), this is actually the behavior of a complex “body” in that space, because numerous “vehicles” (cells and craniofacial organs) must move relative to each other to achieve the correct final configuration (and thus their estimates of positions are constantly changing as the landscape changes dynamically).

Remarkably, this capacity goes beyond the obvious evolutionary advantage of repair from injury to the ability to handle novelty within a creature’s basic components (a capacity that likely improves the evolvability itself). When the cells of a newt are artificially increased in size, fewer of them cooperate to build correctly sized kidney tubules. However, when they are made very large, just one single cell wraps around itself, leaving a lumen to produce the same diameter tubule. In this example, diverse molecular mechanisms (cell–cell communication vs. cytoskeletal bending) are called up in the service of a large-scale state as needed to deal with novel circumstances, including *internal* change. This ability to flexibly harness diverse microstates toward invariant macrostates is a hallmark of multi-scale control in life forms, and the capacity to use different action modules in new ways to achieve a goal is a classic part of many IQ tests. Thus, the collective intelligence of cell swarms operates toward specific goals in the morphospace, able to reach adaptive areas of that space despite diverse starting positions, changing components, or perturbations. All of this can be framed as a kind of behavior in this space, formulating investigations in this field as the search for mechanisms that enable cellular collectives to implement coherent system-level navigation policies.

Creative problem solving (e.g., the reuse of existing affordances in new ways) is revealed most strongly when living systems are pushed well beyond their default configurations by techniques such as chimerism and bioengineering. Skin cells removed from a frog embryo can reboot their multicellularity in a new environment, forming self-motile novel proto-organisms (Xenobots) with numerous capacities, including kinematic self-replication [25,123,124]. These cells reuse the hardware provided by their wild-type frog genome in new ways for coherent morphogenesis, regeneration, and behavior. Thus, evolution produces not only machines that can execute homeostasis for preselected setpoints but highly reconfigurable hardware that is guided by allostasis [125,126,127] and can support diverse goal states. It is no accident that Turing was interested in both intelligence and morphogenesis [128,129], as these problems share a deep invariant.

## 5. 3D Behavior: Movements in Space and Time

**Figure 4:**
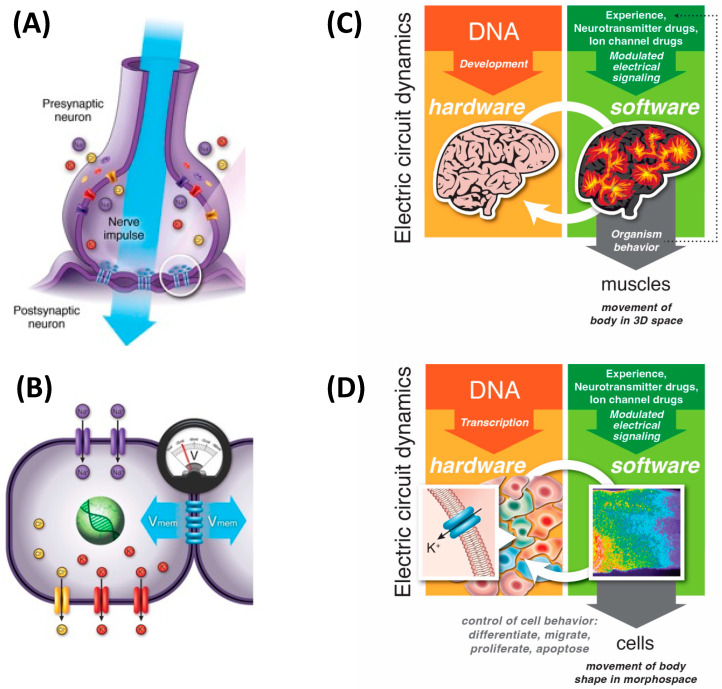
Isomorphism between neural bioelectricity and preneural (developmental) bioelectricity. (**A**) Neural cells compute by forming networks in which each cell can use ion channels to establish a specific resting potential and selectively communicate it to connected neighbors through electrical synapses known as gap junctions. (**B**) Neural dynamics are actually a speed-optimized variant of a much more ancient system. All cells use ion channels, and most cells form electrical connections with their neighbors. (**C**) In the brain, DNA-specified ion channel hardware in neurons enables bioelectric computation, a kind of software that can be impacted by experiences (stimuli). By enabling fast communication over long distances, the synaptic architecture depicted in (**A**) enables the brain to control the physiological dynamics of muscle cells and hence move the body in three dimensions. (**D**) Prior to the development of specialized, high-speed neurons, preneural bioelectric networks exploited the same architecture of physiological software implemented by the same ion channel hardware. The information-processing and memory features of bioelectrical networks were used to control the movement of the body configuration through the morphospace by controlling cell behaviors [104,137]. Images by Jeremy Guay of Peregrine Creative.

Classical behavior in 3D space—what we can call “behavioral space”—is the canonical context in which intelligence is most easily recognized (and the degree of agency is estimated by other observers). It has been proposed (e.g., the skin brain thesis) that behavioral intelligence is the result of increasing demands in coordinating internal sensory-motor organization [6,130,131]. Here, we are proposing an extension of this view in which behavioral intelligence is indeed the product of elaboration of internal computational needs not only of motor coordination but also of morphogenesis. Specifically, we propose that evolution pivoted the strategies used by bioelectric networks to coordinate paths through the morphospace into behavior by simply swapping the sensors and effectors to work in a different space (Figure 4). Indeed, transitional forms exist, showing how morphological control and behavioral control can be implemented by the same system. For example, in the slime mold *Physarum polycephalum* [132,133,134,135,136], which solves problems by growing in specific directions, its motile 3D behavior *is* morphological change. Plants behave the same way, responding to conditions via morphological change.

**Figure 5:**
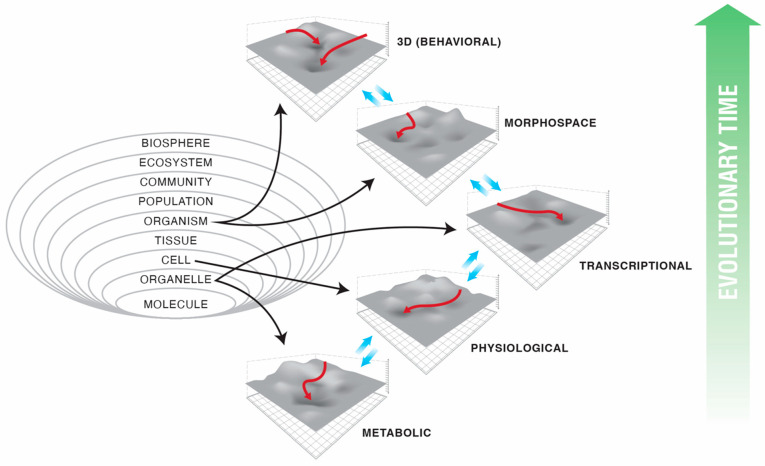
A proposed model in which evolution pivots the same strategies (and some of the same molecular mechanisms) to navigate different spaces. Each level of organization solves problems in its own space, and systems evolved from navigating the metabolic, physiological, transcriptional, anatomical, and finally (when the muscle and nervous systems evolve) 3D space of traditional behavior. Other spaces, such as linguistic space, are possible in more advanced forms. Images by Jeremy Guay of Peregrine Creative.

**Figure 6:**
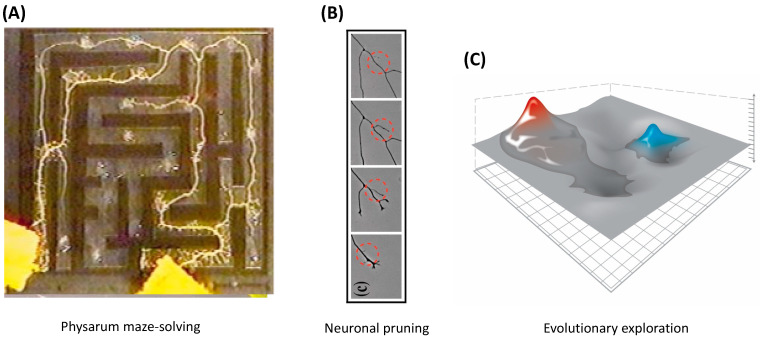
Similar strategies are seen in diverse biological systems at all levels for navigating problem spaces. One example is spreading out and then pulling back from regions that are non-attractors. (**A**) *Physarum* slime molds spreading throughout a maze and then pulling back from every location except the shortest path between two food sources (taken with permission from [144]). (**B**) Neurons often prune back after forming a set of network connections (taken with permission from [145]). (**C**) Evolutionary exploration finds high fitness peaks, and then populations pull back from the valleys. Panel (**C**) by Jeremy Guay of Peregrine Creative.

This pivot (and others like it) across problem spaces (Figure 5) is made possible by three things: (1) network [138,139,140] and probabilistic computation [27,141,142,143] dynamics which are invariant to their material implementation (e.g., Figure 6), (2) modularity of the homeostatic loop, in which the sense, setpoint, and action modules can be swapped out without interfering with the error minimization process, and (3) the fact that evolution, like scientists, is not tied to one specific problem space and is free to pick the perspective from which a problem appears solvable. The development of neural systems borrows heavily from preneural dynamics, utilizing the same molecular mechanisms: ion channels, electrical synapses, and neurotransmitter machinery [35,104].

Important innovations (such as the speed up from minutes to milliseconds and point-to-point connectivity) provide the unique features of each system best suited for its space and the other agents within it. Another important feature of such pivots of molecular machinery into different spaces concerns how they treat space and time. The morphological collective intelligence is primarily concerned with the arrangement of objects in 3D space. The control of movement via nervous systems is largely about events in time. While the brain provides the ability to time travel with respect to behavior (i.e., to remember and plan things that are not occurring right now), there is also preneural bioelectric time travel with respect to a space (encoding pattern memories that serve, for example, as future patterns toward which remodeling and morphogenesis can strive [35,36,117]). For time, the order of arrival, and the sequence response are just edge detection of time, rather than the edge detection in a space performed in morphogenetic events that have to respect compartment borders. The notion of memory is already implicit in homeostatic loops (because setpoints have to be stored) and can be performed by subcellular biochemical circuits [146,147]. Indeed, at least in mammals, episodic memory seems to have evolved from place memory, where the hippocampus and associated areas are mainly place memory in mice and still serve as place memory in humans in addition to encoding episodic memories, which are anchored in space and time. Indeed, recent modeling work suggests that the hippocampus, together with the parahippocampal areas, serves as a general, coordinate-based relational processor [148,149]. Other core elements of cognition—synchronization clocks [150] and space-measuring standards [151]—are equally ancient, perhaps arising from cell cycling or division and distance measuring for cell extension before cleavage (a cytoskeletal task that likely existed in pre-tubulin or actin bacterial cytoskeletal systems).

Even aspects of behavior above the individual organism level are already presaged by preneural dynamics. Multicellularity induces “social” relations at the cellular level. Such relations are prefigured by mechanisms such as quorum sensing [152] in facultative multicellular systems such as microbial biofilms [153]. At the organismal scale, both within- and between-species social relations exhibit complex, context-dependent mixtures of cooperation and competition. Such exchanges are fundamentally communicative, even when they involve destructive interactions such as predation. Hence, they occur in what can be thought of as an informational space [62]. It is natural and, although still controversial, increasingly common to think of information spaces in which innovation, social learning, and intergenerational transfer occur as cultures [154]. Cultures are clearly highly developed in humans via language, the visual and symbolic arts, and the built environment. Since the development of hierarchically organized social life, the resulting information space has ramified into a multi-dimensional virtual reality that includes such cognitive constructs as religion, finance, politics, and science [155]. Evolution in such spaces is fast because, even if one is a crow, memes are cheap [156]. We can expect such memetic evolution to accelerate, in humans and other systems, as additional cognitive prosthetics, including devices usable by nonhuman organisms, are developed.

## 6. Navigating Arbitrary Spaces: A Powerful Invariant

The notion of an action space generalizes from the familiar 3D space of moving behavior to any arbitrary space of some number of dimensions, within which an agent can act. Hence, all of the specific spaces discussed above can be considered components of an organism’s action space. These spaces are constructs created by the system itself to organize its activity and make sense of its world and by an observer (e.g., a scientist or a conspecific) who constructs a filter with which to be able to understand and predict the actions of the agent. A most useful thing about spaces is that they serve as a central symmetry between phenomena such as classical behavior, physiology, metabolism, gene expression, and morphogenesis. They are a unifying principle, an invariant that fundamentally defines what an active agent is and how they can be recognized, compared, and manipulated.

In fact, there are actually two symmetries here. One is scale: the fact that the same type of dynamic is acting at the molecular, cell, tissue, organism, and community scales. The second is across spaces: the same strategy can be pivoted by evolution to explore behavioral space after it is honed, exploring metabolic, transcriptional, and morphospaces. The high degree of conservation of mechanisms and algorithms between, for example, morphology and cognition is an example of this symmetry across the course of evolution [35,104,157]. All of these unconventional agents navigate their spaces with various degrees of competency, enabling all of the tools created to understand and control the traversal of spaces in animals and machines to be deployed on a very wide range of problems.

The notion of action spaces shows how to connect goal-directed activity to notions of energy (e.g., of evolution toward an attractor). For biological systems at any scale, the relevant attractors are those that implement allostasis within the current environment. The variational free energy (VFE) principle formulates this requirement for allostasis in information-theoretic terms: living systems behave so as to maximize their ability to predict their own future states. When we identify VFE with uncertainty and hence with (the probability of) prediction error, the minima of VFE become the maxima of predictive success. Uncertainty, and hence VFE, is distinct from metabolic load. Hence, we can ask about the metabolic cost of achieving an increment of predictive ability. The metabolic cost of generating predictions through the use of some computational model of the environment emphasizes that moving through the search space really is a *search*. It takes effort and resources, including memory resources. As cells or other systems become stressed (e.g., by lack of sufficient free-energy resources), their generative models can be expected to deteriorate toward stochastic defaults, and hence their searches can be expected to deteriorate toward random walks. This has been observed at the cellular level [158] and is a commonplace observation in stressed organisms, including humans.

The act of navigating a state space, whatever its degrees of freedom, involves several fundamental components. First, there is the inverse problem of which effectors to activate to reach a preferred region of state space. It frames most aspects of survival as, fundamentally, a search with different degrees of capacity to look into the future. Second, it is greatly potentiated by the ability to maintain a record of the past (i.e., a reliable, readable memory). The central question faced by any system is what to do next among certain choices. Thus, it is important to begin to formalize a notion of decision making in a deterministic system. No finite agent can discover all of the causal influences that determine its own behavior, as global determinism logically disallows local determinism. Hence, global determinism assures “free will” from every (finite) local perspective [159]. The failure of theories modeling human decision making on what human agents can consciously report about their thought processes makes the need for a general theory evident [160,161]. The mathematical theory of active inference as Bayesian satisficing provides a scale-free framework for understanding this process. Given that spaces are an essential invariant for understanding biological adaptive activity, it is important to ask how these spaces originate.

## 7. Active Inference Generates Spaces

### 7.1. Organisms Interact with Their Environments via Markov Blankets

Biological systems at all scales exist and maintain their integrity in active exchange with their environments. As first shown by Ashby [162], this exchange can be formalized as an exchange of information. From this formal perspective, organisms act so as to minimize the VFE of their interactions with their environments. While the FEP was originally formulated as a theory of brain function [163,164,165], it has since been applied to a wide range of biological systems and processes [27,142,166,167,168] and was recently shown to characterize any classical [169] or quantum [170] system that is sufficiently stable to be identifiable over macroscopic time. Allostasis is, in other words, not limited to biology; it is a general characteristic of all systems that resist the entropic forces of their environments long enough to be observed at multiple times. Indeed, the emergence of the structural complexity required to sustain allostasis can be seen as being driven by the environment as a means of producing entropy [171].

Maintaining allostasis is maintaining a distinction between “my states” and “my environment’s states,” where “my environment” here is everything other than me. A *state* is a collection of values of some degrees of freedom, or *state variables*. Position, temperature, viscosity, concentrations of various molecules, and electrical charge are all state variables of relevance to all organisms. The internal-external distinction can be formalized in terms of a *Markov blanket* (MB), a set of intermediate states that serves as an interface between inside and outside [27,172]. These interface states transfer information from outside to inside (i.e., implement perception) and from inside to outside (i.e., implement action). An organism and its environment share, by definition, the same MB; they merely “look at” different sides of it. *Position* states on a MB implement the “physical boundary” of an organism, with the cell membrane or the skin as examples. This boundary is, from the organism’s perspective, also the physical boundary of its environment. Most MB states encode values of variables other than the position (e.g., photon intensity and frequency (brightness, color, radiant temperature, etc.), air pressure (e.g., wind velocity and sound), or molecular concentrations (e.g., osmolarity, smell, and taste)).

**Figure 7:**
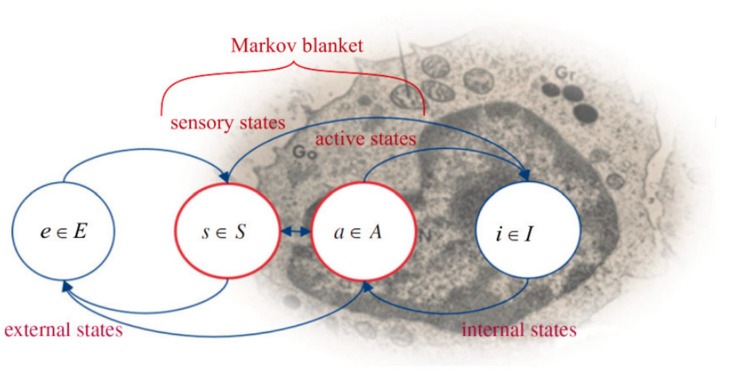
Schematic specifications of a Markov blanket (MB) comprising sensory (s) and active (a) states that are intermediate between the external or environmental (e) states and the internal (i) states of some system of interest. The MB is a boundary in the joint system-environment state space. It may be partially implemented by a structure (here, a plasma membrane) in a 3D space. The MB states (s and a) can be thought of as an API between the system and its environment. Taken from [157] and used with permission.

As *all* information exchange between a system and its environment passes through the MB, an organism’s perceptions “of its environment” are, from a mechanistic perspective, data encoded by its environment on its MB (Figure 7). An organism’s actions “on its environment” are, similarly, data it encodes on its MB. Indeed, we can view an organism’s actions as its environment’s perceptions and vice versa. An organism has no access, even in principle, to the mechanisms by which its environment encodes data on its MB. This restriction on access is fully symmetrical; the organism’s environment has no access to how the organism encodes data on the MB. (Indeed, an organism is, by definition, “the environment” of its environment.) While such statements are sometimes considered “anti-realist” or “subjectivist” [173], they are just consequences of modeling physical interaction as information exchange [174,175]. Organisms such as humans that employ technologies to extend their perception and action capabilities are, effectively, extending their Markov blankets to encode the values of additional state variables.

When information flow is restricted by an MB, the task of minimizing the prediction error and hence minimizing the VFE becomes the task of predicting and then acting to regulate the future state of the MB. The good regulator theorem [176] requires any system capable of such regulation to be or to encode a *generative model* [27] of its environment’s actions on its MB. The state variables of this model are the state variables of the MB, the only state variables that can be either measured or predicted. The generative model encoded by an organism is thus the organism’s “theory” of its environment’s observable behavior (i.e., its environment’s actions on its MB) and includes, most importantly, its theory of how its environment will respond to each of its own actions on its MB.

It is important to emphasize that, as shown in [169,170], these considerations apply to all physical systems at all scales. While here, we will be concerned primarily with individual organisms, Markov blankets as system–environment interfaces and VFE minimization as an inferential mechanism characterize all systems identifiable as such over time, including macromolecules, biomolecular pathways, individual cells (whether free-living or components of multicellular organisms), organs and tissues, individual organisms, communities of organisms, ecosystems, and even larger structures. Indeed, the authors of [177] showed how to model the global climate system by minimizing the VFE across an MB. We can, therefore, consider MBs to be *universal*, *scale-free structures* and VFE minimization to be a *universal*, *scale-free mechanism*. Hence, MBs and VFE minimization are invariants that characterize all forms of behavior in all “spaces” occupied and explored by organisms.

### 7.2. Behavioral “Spaces” Are Tractable Components of an Overall State Space

We are now in position to define the spaces in which an organism operates. Suppose an organism’s MB encodes at most *m* distinct values of each of *n* distinct variables, and let *N* = *nm*. This number N is finite for any finite system (i.e., any system with finite energy resources and hence a finite measurement resolution). Any state of the MB can then be considered to be a vector in an *N*-dimensional vector space constructed by assigning a basis vector to each of the *N* variable-value combinations and adopting the standard notion of distance between vectors as a metric. Such vector spaces are called *Hilbert spaces* and are widely used in quantum theory. They can also be employed in classical physics. Any organism—indeed, any physical system, classical or quantum—can be considered to behave in the Hilbert space that characterizes its MB. A perception-action loop is, in this formalism, simply a mapping from an “input” vector representing the state of the MB at some instant *t* to an “output” vector representing the state of the MB at some later instant *t* + Δ*t*. As this is a well-behaved map between vectors, it can be treated as linear *independent of its implementation*. It is this “hiding” of implementation details that renders MBs such useful theoretical tools. They can be thought of as defining application programming interfaces (APIs) around physical systems that specify the data structures that interactions must respect. The consequences of this are discussed further below.

**Figure 8:**
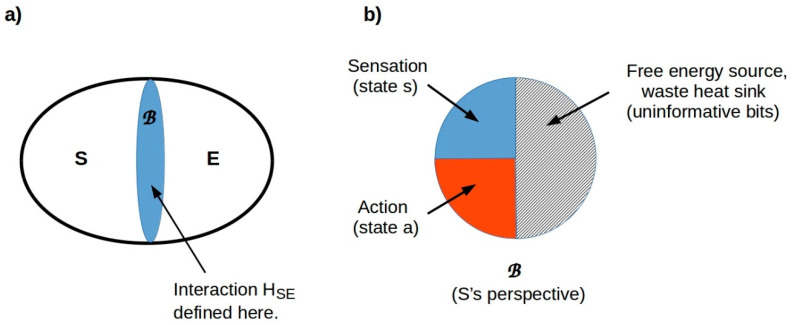
Generic two-system interaction mediated by a Markov blanket. (**a**) Any MB can be considered a boundary B in the joint state space of a system S and its environment E. The physical interaction between S and E, here represented by the Hamiltonian (total energy) operator H_SE_, is defined at this boundary. (**b**) S must obtain free energy from and exhaust waste heat into E. The boundary B must therefore include a thermodynamic sector in addition to the sensory (s) and active (a) sectors. The states of this thermodynamic sector are observationally inaccessible and hence uninformative to S. Taken from [78] with a CC-BY license.

For any biological system, the number *N* is enormous, and the complexity of a predictive (generative) model of an *N*-dimensional space increases combinatorially with *N*. Hence, organisms cannot be expected to implement full, predictive models of the Hilbert spaces of their MBs. Indeed, a model of the full Hilbert space of the MB is impossible in principle. The MB is, by definition, the sole interface in the joint system–environment state space between any system and its environment. Hence, some fraction of the states of the MB of any finite system must be allocated to free energy acquisition and waste heat removal [178]. The sector of the Hilbert space allocated to these thermodynamic functions is observationally inaccessible to the system; its sole function is a thermodynamic one. Hence, the MB of any finite system can be regarded as divided into at least three sectors, comprising sensory, active, and thermodynamic states as shown in Figure 8.

Because MB states cannot be modeled completely, organisms, including humans, instead implement partial models of sets of variables that have been observed to covary systematically. The positions of objects, for example, covary as an organism moves. A model that captures this covariance is a model of an ordinary 3D space. Concentrations of environmental chemicals also tend to covary; the space of chemical concentration gradients is the primary “space” in which chemotactic microbes operate [179] and is an important space for all organisms equipped with olfaction and taste. Organisms can, in general, be expected to optimize the use of their limited information-processing resources by limiting their generative models to just the principal components of their experience and segmenting these models into “spaces” spanned by covarying principal components.

Segmentation of an overall state space into predictively tractable subspaces is greatly facilitated by the fact that tractable subspaces tend to exhibit relatively simple symmetries. The most familiar are the translational, rotational, and relative motion symmetries of objects in a 3D space. Moving an object in a 3D space does not modify its properties or change its identity. These symmetries are described mathematically by the Galilean group in classical physics and by the Poincaré group when special relativity is taken into account. Hence, a generative model of spacetime is, at minimum, a representation of the Galilean or Poincaré groups. State variables that can be described by fields in spacetime (e.g., electric or magnetic fields) satisfy gauge symmetries, which prevent the state of the field from depending on how it is measured. Using a quantum theoretic framework, it can be proven that any state variables encoded on a Markov blanket must satisfy the gauge symmetries if they are represented as a field in spacetime [180]. See [143] for an application of gauge-theoretic ideas in neuroscience.

Symmetries create redundancy, and many different descriptions of a symmetric situation encode the same information. This redundancy enables data compression and coarse graining. It also makes any space characterized by symmetries an error-correcting code. Information that may be missing or ambiguous at one “location” in the space can be found at other locations [181]. Redundancy enables “babbling” as a strategy for discovering the symmetries of a space. The language and motor babbling of infants are a canonical example. Babbling can be considered a heuristic search strategy in which “random” actions are deployed to investigate the large-scale structure of a space, and more directed minor variations of “interesting” actions are used to investigate the local structure (for comparisons of human infant and developmental robotic implementations of babbling, see [182,183]). Such alternation between breadth-first and depth-first searching in a space is an ancient strategy of living systems, going back at least to the run-and-tumble behavior of chemotactic bacteria. The same strategy (in effect, babbling in a 3D space instead of in a linguistic space) has been shown to be very effective in robotics, enabling the building of adaptive robots that develop models of themselves and strategies to navigate the world de novo [184].

### 7.3. Problem Spaces Are Observer-Dependent

Problem spaces are defined by observers, as they make models to help explain, predict, and control other systems. Crucially, however, the system itself is also such an observer [185] and generates models of spaces to help guide activity. In humans, the personal past is such a space, as increasingly more detailed studies of the construction of episodic memories demonstrate [186,187]. A very fundamental way for even simple observers to generate the notion of spaces ab initio is from the commonalities between actions required to nullify changes in sensory experience. Actuations that result in predictable changes in sensory states can often be naturally represented as “movement” in a space [188]. As discussed above, an obvious and well-studied example is “babbling”—both vocal and motor—in human infants, a phenomenon also common in other animals [189] and increasingly employed in developmental robotics [182,190]. Such space construction is closely linked to the very basal capacity for homeostasis (keeping a sensory state in a constant range is just one step past keeping a specific variable, such as the pH level, in the right range), but this may involve feedback and monitoring of the result of one or more layers of processing past the raw sensor. This loop immediately provides the opportunity to scale intelligence via optimized user illusions (models) of spaces [181,183,191,192] because there are many levels of sophistication available to the overall project of keeping one measurable optimized by taking various actions. This scheme is not only about actuating muscle or ciliary motion to keep a constant relationship with a spot of light, for example; it works in other spaces, too. The barium-exposed planaria are looking for moves in transcriptional space that allow them to keep their normal physiological states. Similarly, somatic tissue develops and monitors representations of its own anatomical layout, using bioelectrics in epithelia as a kind of “retina” that perceives the body structure [193,194] and is able to trigger movements in the morphospace to counter the induction of incorrect layouts in the large-scale morphospace (such as the fact that tails grafted onto inappropriate locations in amphibians will become remodeled into limbs, a structure more appropriate to the new *location* [195,196]). When brains developed, they retained and amplified the notion of modeling the self in anatomical space via the somatotopic homunculus [197].

### 7.4. Tractable Spaces Correspond to Perception and Action Modules

Separating the overall (Hilbert) state space of the Markov blanket into predictively tractable components has the effect of breaking the overall prediction problem—the problem of minimizing VFE—into tractable components. As these component problems involve spaces with, in general, different symmetries, the most efficient methods of solving them will, in general, be different. They can each, in particular, be expected to involve data structures (“representations”) that encode the symmetries of the relevant space. These data structures are, in turn, encoded by the Markov blanket. Formally, they are the basis vectors of the corresponding Hilbert space. Maximizing efficiency (i.e., minimizing the resource requirements of information processing) requires that perception and action both employ the same data structures and hence respect the same symmetries. Hence, perception and action in any domain can always be viewed as acting via a particular, domain-specific component—a particular subspace with its own basis vectors—of the overall Markov blanket.

A perception–action module that imposes a particular data structure can be considered to define a *reference frame*, and when such a module is physically implemented by a finite system that consumes energy and dissipates heat, it becomes a quantum reference frame (QRF) [197,198] (see [170,199] for discussion in a biological context). The most familiar QRFs are artifacts, such as meter sticks or clocks, that we humans use to make external measurements. Employing such artifacts to measure distance and time, however, requires an internal sensory representation of distance and time. A person with no ability to sense duration, for example, could make no sense of a clock [200]. Hence, biologically implemented QRFs underlie the use of all artificial QRFs. Any pathway that employs a fixed (or only slowly varying) reference point (e.g., the midpoint of a sigmoid activity curve) to switch some behavior on or off can be considered a QRF. The use of the [CheY-P]/[CheY] concentration ratio to control the direction of flagellar motion in chemotactic bacteria provides an ancient example.

A perception–action module can compare perceptions and hence regulate actions only over the timeframe of its local memory. Maintaining a local memory requires energy. The [CheY-P]/[CheY] ratio, for example, is maintained by enzymatic activity and hence by metabolic activity. Selective pressure to minimize VFE is, therefore, selective pressure to expand the memory capacity (i.e., to allocate increased structural and energy resources to storing information about the consequences (in context) of past actions). As obtaining additional resources from the environment may require sensing and acting on the environment in new ways—from predation to social or economic exchange—VFE minimization can be expected, in general, to drive the development of new QRFs, with the elaboration of progressively more complex visual, auditory, and olfactory systems in lineages subject to different selection pressures as obvious examples. Increasing the information processing capability is, therefore, inevitably a positive feedback loop and hence effectively an arms race with selection pressures from the environment.

The Heisenberg uncertainty principle famously limits the simultaneous use or *co-deployability* of some pairs of QRFs (e.g., those for position and momentum) at high measurement resolutions. Interference between the measurements of degrees of freedom assumed *a priori* to be independent, generically termed *context effects*, can be generated even in classical systems [201] and can always be attributed to failures in commutativity (i.e., interference) between QRFs [202]. Competition for energetic resources between QRFs also limits co-deployability. Systems respond to limits on co-deployability by developing attention systems that prioritize both perceptions and actions. By serving as a resource allocation mechanism, attention itself becomes a resource.

### 7.5. Experimentally Probing a System’s QRFs

When we perform experiments on a system, we are acting as part of that system’s environment. Our actions on the system and our measurements of its behavior depend on our QRFs and hence on the spaces in which we operate. Our inferred explanations of the system’s behavior become components of our generative models, which we test by testing their predictions.

How can we, from this position outside of the system’s Markov blanket, determine what QRFs the system is deploying and hence determine the spaces in which it operates? It is clear from the definition of a Markov blanket and a generic result of quantum information theory [178,203] that no such experimental determination can be made. The best that can be accomplished is an empirical model of the system’s QRFs and hence of its operating spaces, developed within the language imposed by the experimenter’s QRFs. Even biochemical pathways, from this strict perspective, are theoretical models based on evidence that may be limited or ascertainment-biased by the experimental procedures employed. The science of QRFs is, in other words, subject to the same fundamental limitations of any other science. It is greatly facilitated by building models of the system of interest as embedded in and communicating with its environment and then examining these models at multiple scales. The mammalian hippocampus, for example, functions in part as a spacetime QRF at the scale of the whole organism but functions as a pulse correlation generator at the scale of the local networks to which it supplies inputs [204].

From an operational perspective, probing a system’s QRFs is an exercise in reverse engineering, inferring a “design model” that meets the goals of functionality and efficiency from experiments that probe structure (to the extent that it is observationally accessible) and overt behavior. Inferring the representations and hence the data structures employed by an organism to process and act on information from its environment is, effectively, inferring the API of a computational system for which only the input-output behavior and external resource usage are initially known. While a recognizable hardware architecture can contribute useful information to this process, it places few if any constraints on the software architecture and hence on the structure of the API. The use of experimental methods modeled on those of cognitive psychology is the present state of the art for reverse engineering the functions implemented by deep learning systems following training [205]. We may increasingly expect the same to be the case in biology.

### 7.6. Common Inference Mechanisms Induce Symmetries between Spaces

All complex biological systems are hierarchical; macromolecules are organized into larger-scale structures and pathways, which are organized into functioning cells, which form tissues, organs, and eventually whole organisms, which are then organized into societies and ecosystems, etc. A crucial aspect of this is that the hierarchy (modularity) is not simply structural. Each level contains its own agency, with agendas in various appropriate spaces. The robustness and plasticity of life may be due to the unique and powerful ways in which the lower levels’ activities (microstates) are harnessed toward the higher levels’ goals. Agents at higher levels (e.g., organs) deform the energy landscape of actions for the lower levels (e.g., cells or subcellular machinery), such as the example in Figure 3I. This enables the lower systems to “merely go down energy gradients”, or to perform their tasks with minimal cognitive capacity while at the same time serving the needs of the higher-level system, which has exerted energy (via rewards and other actions) to shape its parts’ geodesics to be compatible with its own goals. This is a very powerful aspect of multi-scale competency because the larger system does not need to micromanage the actions of the lower levels; once the geodesics are set, the system can *depend* on the lower levels to do what they do best: go down the energy gradient. The paths of least action in any space are implemented by the paths of least action (i.e., VFE-minimizing paths) in the overall manifold of the internal state probabilities. Recent work in Bayesian predictive processing shows how an agent’s information geometry is distorted by beliefs, a kind of gauge theory [143] that tightly links the notion of action in an arbitrary space to the cognitive state of the agent as an invested observer. For example, the meta-cognitive level of attention can be seen as setting the precision (curvature of the free energy) for another part of the internal model. It is this set of multi-scale relationships, with parts deforming subparts’ action spaces toward goals in their own space, that distinguishes flat (single-level) systems that simply minimize energy (e.g., water flowing down a hill, which people do not think of as an action or decision) from ones that use the same kind of physics in a more obviously cognitive manner.

As noted above, systems at every level in such hierarchies can be described as performing active inference with the goal of minimizing environmental VFE, where the environment is everything other than the system. Systems at any hierarchical level can, in other words, be considered to deploy generative models of the behaviors of their environments to interpret what they perceive and to employ these same models to act on their environments in return. Biological hierarchies are, moreover, not just structural; they are also functional. How do actions or functions at one level affect the actions or functions at other levels? It is to this question that we now turn.

**Figure 9:**
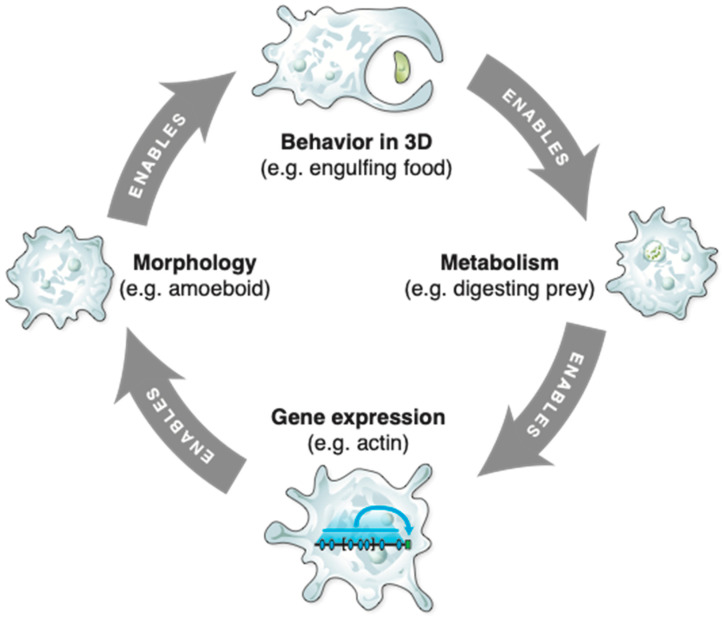
Actions in one space enable (or constrain) actions in another space. These relations function both from the bottom up and the top down in the scale hierarchy. Gene expression, for example, provides the components needed to enable a particular morphology, which in turn enables behaviors that enable the free energy production required to drive further gene expression. Enabling and constraining relations function, in general, both from the bottom up and the top down in the scale hierarchy. Image by Jeremy Guay of Peregrine Creative.

Consider an amoeboid cell. Actions in the macromolecular state spaces that define the genome, transcriptome, and proteome (e.g., expressing an actin gene) enable actions in the morphological space (e.g., pseudopod extension), as shown in Figure 9. The macromolecular actions are carried out by macromolecular complexes, in this case transcription, mRNA processing, and translation systems. The morphological actions that they enable are carried out by much larger-scale structures, in this case spatially organized associations between the cytoskeletal components and mitochondria. These organelle-scale actions in turn enable cellular scale actions such as environmental exploration and predation. Bottom-up enabling relations such as these have top-down counterparts; predation enables metabolism of prey components to yield usable free energy, which in turn enables macromolecular actions such as gene expression.

## 8. Implications: A Research Program

We have shown here how MBs and VFE minimization define mutually interdependent behavioral spaces at multiple scales and then organize behavior to maximize predictability, hence maximizing the probability of continuing allostasis within those spaces. This analysis leaves open, however, the question of how either MBs or VFE minimization are implemented in the vast variety of biological and increasingly hybrid biological and artificial systems to which we have experimental access. Hence, there remains a large number of further areas for conceptual development as well as empirical capabilities that should be investigated, which include the following.

### 8.1. Conceptual Questions and Further Links to Develop

- While higher-level systems bend action spaces for lower-level subsystems, it can be predicted that the higher level no longer needs to operate in a very rugged space of microstates. Instead, evolution can search a coarse-grained space of interventions, which also includes changing the resource availability landscapes at both the lower and higher levels (e.g., inventing a mouth and a specialized digestive system). Computational models can be created to quantify the efficiency gains of evolutionary search in such multi-scale competency systems.
- Links can be made to higher levels of cognitive activity and neuroscience. For example, yoga and biofeedback can be seen as ways for systems to forge new links between higher- and lower-level measurables. Gaining control over formerly autonomic system functions is akin to rerunning causal analysis functions on oneself to discover new axes in physiological spaces that the higher-level self did not previously have actuators for. Such processes clearly depend on interoception, a process for which active inference models are now well-developed [206,207], and being integrated with models of perception in a shared memory global workspace architecture [208].
- More broadly, models of space traversal help flesh out a true continuum of agency, placing simple systems that only know how to “roll down a hill” on the same overall spectrum as psychological systems that minimize complex cognitive stress states. Concepts related to free energy help provide a single framework that is required to explain how complex minds emerge from “just physics” without magical discontinuities in evolution or development. The capacity to traverse a space without getting caught in local optima can be developed into a formal definition of IQ for a system in that space. This links naturally to the work in morphological computation and embodied cognition because body shape determines the IQ of traversing a 3D behavioral space. How does this extend into other spaces? Many fascinating conceptual links can be developed to work on embodied premotor cognition in math, causal reasoning, general planning, etc. [209,210,211].
- How do cells, both native and after modification via synthetic biology tools, make internal models of their “body shape” in unconventional spaces, such as a transcriptional space? Cells in vitro can learn to control flight simulators [212], as can people with BCIs [213]. Brains can learn to control prosthetic limbs with new degrees of freedom [214]. What self- and world-modeling capacities are invariant across such problem spaces?
- The tight link we have developed between motion in spaces and *degrees* of cognition across scales suggests that it may be possible to develop models of evolutionary search itself as a kind of meta-agent searching the fitness space via active inference and other strategies [62,63,215,216]. In this light, evolution is still not claimed to be a complex meta-cognitive agent that is knowingly seeking specific ends, but on the other hand, it may not be completely blind either. It may be possible to develop models of minimal information processing that better explain the ability of the evolutionary process to solve problems, to choose which problems to solve, and to give rise to architectures that not only provide immediate fitness payoffs but also perform well in entirely new environments.
- A key opportunity for new theory concerns what tools could be developed for a system to detect that it is part of a larger system that is deforming its action space with nonzero agency. It may not be possible due to the Gödelian limits for a system to fathom the actual goals of the larger system, of which it forms a part, but how does an intelligent system gain evidence that it is part of an agent with some “grand design” versus living in a cold, mechanical universe that does not care what the parts do? The Lovecraftian horror of catching a glimpse of the fact that one is a cog in a grandiose intelligent system may be tempered by mathematical tools that enable us to have more agency over which aspects of the externally applied gradients we wish to fight against and which gradients we gladly roll down.
- We foresee great promise in the application of the mathematical framework of category theory [217,218], which provides the conceptual and formal tools needed to model the relationships between arbitrary spaces. Any of the spaces discussed here, together with the search operations acting within that space, can be considered a category. The theory provides, in this case, rigorous tools for determining whether multiple paths through the space yield the same outcome and, even more interestingly, whether paths through different spaces, such as a path in a morphological space or a path in a 3D behavioral space, yield the same outcome. We defer such analysis to future work. Some preliminary steps in this direction, characterizing arbitrary QRFs as category-theoretic constructs, can be found in [170,178].
- There are numerous analogies to be explored with respect to porting conceptual tools from relativity to study scale-free cognition. The use of cognitive geometry and infodesics [219] ties naturally to general relativity. Other examples include the following:
  ○ Gravitational memory (permanent distortions of spacetime by gravitational waves [220]) to link the structure of action spaces to past experience;
  ○ Inertia in terms of resilience to stress (anatomical homeostasis as a kind of inertia against movement in the morphospace and other spaces);
  ○ Acceleration and force in a network space, where every connection in a network could be modeled via a “spring constant” or, even better, an LRC circuit. With feedback, interesting oscillations can appear, which can be harnessed as computations;
  ○ The ability of one system to warp the action space for another, such as warping the morphospace for the embryonic head by specific organ movements, generates an analog of “mass”;
  ○ Bioelectric circuits could be modeled as warping the morphospace in the same way wormholes warp physical space. The two points at opposite ends of a wire are, for informational purposes, the same point, even if they are on opposite sides of the embryo. Neal Stephenson stated, “The cyberspace-warping power of wires, therefore, changes the geometry of the world of commerce and politics and ideas that we live in’’ [221]. The gap junctions’ control of morphogenetic bioelectric communication deforms the physiological space to overcome distance in the anatomical space. Neurons do this too, as do mechanical stress in connective tissue and hormones;
  ○ Links also could be made to concepts of special relativity. For example, doppler effects in morphogenesis have already been described [222]. Moreover, the limited speed at which information can propagate through tissue naturally defines a minimal “now” moment, a temporal thickness for the integrated agent below which only submodules exist, in effect illustrating the relatedness of space and time by the propagation speed of information signals within living systems.

### 8.2. Specific Empirical Research Directions

- Specific models of morphogenetic control (embryogenesis, regeneration, cancer, etc.) that rely on navigation policies with diverse levels of cognitive sophistication need to be created and empirically tested. Can craniofacial remodeling be understood as a “run and tumble” strategy? Can evolution of morphogenetic control circuits be understood as the evolution of abstract vehicle navigation skills, thus porting knowledge from evolutionary robotics and collective intelligence to developmental biology [157,223,224,225]?
- Similarly, such models need to be developed to understand allostasis in transcriptional, metabolic, and physiological spaces, modeling and then developing minimal Braitenberg vehicles [44,226,227,228,229] as real devices to implement biomedical interventions such as smart insulin and neurotransmitter delivery devices.
- Regenerative medicine needs to be moved beyond an exclusive focus on the micro-level hardware (genomic editing and protein pathway engineering) to include interventions at higher levels. Using tools from behavioral science such as training in various learning assays can manipulate the lower-dimensional and smoother space of tissue- and organ-level incentives (described in more detail in [35,36]). Much as evolution exploits multi-scale competency to maximize the adaptive gains per change made, bioengineers and workers in regenerative medicine can take advantage of behavior shaping of cellular agendas and plasticity, working in a reward space. Interestingly, this was well-appreciated by Pavlov, whose early work included training animals’ *organs* in addition to the animals themselves. He understood the physiological space, and his experiments on training the pancreas and other body systems can now be performed with much higher-resolution tools. More broadly, impacting and incentivizing decision-making modules at higher levels is much more likely to produce coordinated, coherent outcomes than interventions at lower levels [230], resulting in fewer side effects in pharmacology and avoiding unhappy monsters in synthetic bioengineering. The future of biomedicine will look much more like communication (with unconventional intelligences in the body) than mechanical control at the molecular pathway level. This includes signaling to exploit the control policies of cells in the morphospace for regenerative control of growth and form [35,36] and exploiting gene-regulatory networks’ abilities to learn from experience to modify how they move in the transcriptional space while healthy and in the case of disease [79,83,231,232,233,234,235].
- Computer engineering and robotics also afford many opportunities for testing and applying this framework. Incorporating biological concepts into a computing system design has been explored in the abstract [236,237], at the level of system design [238,239], and with neuromorphic hardware [240,241]. The present work suggests further directions, including developing frameworks for working with agential materials (like the cells that make up Xenobots), which requires distinct strategies from those used with passive materials or even active matter [242,243,244,245], creating evolutionary simulations and human use tools to explicitly address multiple scales of organization and problem solving.
- More broadly, artificial intelligence can benefit from enhancing current neuromorphic approaches with systems based on much more general, ancient intelligence, creating systems with motivation and agency from the ground up by taking embodiment seriously from an evolutionary perspective. The classic Dennett and Minsky debate about how much real-world embodiment matters for artificial intelligence can now be reframed in more general terms: embodiment is critical indeed, but it does not have to be in the classic 3D space. Embodiment in other action spaces can drive the same intelligence ratchet described above. New general AIs are likely to be developed gradually from minimal systems driven by the dynamics described above, which eventually scale homeostatic action into advanced metacognition. One specific strategy that can be suggested is the creation of an unsupervised agency estimator, which seeks to make models of its environment anywhere on the spectrum of persuadability [9]. This system will not only be useful for human scientists (freeing their hypothesis-making from the mindblindness [246] that limits imagination with respect to unconventional intelligences); it can also be used in an “adversarial” mode with evolving intelligences, a cycle that increasingly potentiates both the intelligence and the ability to detect it.

## 9. Conclusions

Human beings evolved with conscious access mostly to data from the outside world, including sensitivity to only a very small slice of the myriad of actions occurring in their various physiological, cellular, metabolic, and morphogenetic control systems. As a result, while our cognition is finely tuned to identify levels of agency in the behavioral actions of objects moving in a 3D space, we are intrinsically bad at recognizing intelligence in unfamiliar guises. If, for example, we grew up with a keen internal sense of our blood chemistry and all the things our pancreas, liver, kidneys, etc. were doing to maximize our health, or if we could directly sense changes in gene expression, we would have no trouble recognizing these as agents exhibiting competency and degrees of intelligence in other spaces. Thus, it is essential to develop a substrate- and scale-invariant theory of agency and intelligence. Specifically, we have sketched such a theory while maximizing the empirical, testable, and practical implications over philosophical wrangling. We have shown in particular that living systems operate in multiple spaces at different scales. These include the transcriptional, physiological, and morphological spaces as well as the more familiar 3D behavioral and social spaces. The computational mechanism of VFE minimization drives behavior in these spaces toward attractor states that enable allostasis. This framework suggests a number of new theoretical and experimental approaches in both biological and hybrid biological-artificial systems.

Our model is committed to an observer-dependent non-binary approach that takes evolution and developmental biology seriously to emphasize gradual origins, deep unification of basic principles, and ubiquitous real-time change. Policies and mechanisms guiding such seemingly diverse behavior as magnets’ “mindless” movements to reach each other, biochemical networks moving down energy gradients, bacteria swimming up nutrient gradients, moths’ repeated attempts to reach a light, and human goal-directed behavior *must* be on the same continuum because modern biology offers no discrete magical event that separates them; a single framework is needed. In this light, all intelligences are collective intelligences, and biological systems are nested dolls of agents with agendas that cooperate, compete, communicate, and interact within and across levels of organization. Agents of highly diverse implementation model their environments and themselves in accordance with an active inference framework, which drives the way they navigate information spaces. While dynamical systems theory describes how a system can go through a defined space, multi-scale agency models explain the shape of the space relative to specific observers and agents [247].

A key aspect of life is top-down control, where higher levels deform action spaces so that lower levels can be less intelligent and more mechanical while enabling the larger system to occupy the more adaptive regions of various spaces. The system evolves via a ratchet mechanism that begins with simple homeostasis and takes advantage of its modularity to measure, remember, and act over progressively larger and more complex states. Evolution pivoted this basic trick across spaces from the metabolic and physiological spaces through the anatomical morphospace, where ancient bioelectric network mechanisms became used to propel animals through a 3D space using the same strategies they originally relied on to move their anatomical configuration through morphospace during regulative development and regeneration.

The “brain in a vat” dynamic, where agents have no access to the objective ground truth about their brain–body–environment relations but must build actionable models of themselves on the fly, helps understand (and design) highly diverse systems in which all of these aspects can be changed in a modular way: by evolution and by engineers. This helps clarify why biological systems are so highly evolvable and dissolves the existing concepts of objective privileged viewpoints of boundaries between the self and the world. These ideas have numerous implications for understanding the origin of various control systems in neuroscience and the efficiency of evolution in creating extremely robust problem-solving machines. This in turn suggests the same kinds of strategies (targeting high-level reward spaces) that are useful for workers in biomedicine, AI, and synthetic bioengineering who seek to manipulate and build complex adaptive systems.

We view this framework as only the beginning of an empirically grounded understanding of agency that provides a conceptually integrated picture of the world. It is crucial to develop such frameworks that abandon untenable binary distinctions in favor of predictive, actionable models that are compatible with the modern understanding of gradual evolution and developmental biology. Forthcoming advances in synthetic bioengineering and AI will result in a diversity of agents in our midst that will dwarf Darwin’s challenge to classical categories. Future developments in transformative regenerative medicine, automation, and ethics require a firm conceptual foundation for understanding agency in a physical world.

## Data Availability

Not applicable.
